# The associations between the response efficacy and objective and subjective change in physical activity and diet in the Information and Risk Modification trial

**DOI:** 10.1016/j.puhe.2018.09.006

**Published:** 2018-12

**Authors:** C. Wou, B. Silarova, S. Griffin, J.A. Usher-Smith

**Affiliations:** a The Primary Care Unit, Department of Public Health and Primary Care, University of Cambridge School of Clinical Medicine, Box 113 Cambridge Biomedical Campus, Cambridge, CB2 0SR, UK; b MRC Epidemiology Unit, Institute of Metabolic Science, School of Clinical Medicine, University of Cambridge, Box 285 Cambridge Biomedical Campus, Cambridge, CB2 0SL, UK

**Keywords:** Response efficacy, Behaviour change, Physical activity, Diet, Premature mortality, Prevention

## Abstract

**Objectives:**

Many health promotion campaigns and interventions focussing on improving health-related behaviours have been based on targeting response efficacy. This is based on the assumption that response efficacy is an important modifiable determinant of behaviour change. This study aimed to quantify the association between response efficacy and objective and subjective measures of physical activity and diet.

**Study design:**

Prospective cohort analysis of data from a randomised controlled trial.

**Methods:**

A total of 953 participants were assessed for response efficacy at baseline and 12 weeks following randomisation to interventions to increase physical activity and improve diet. Subjective measures were collected via a self-report questionnaire that included two questions used to derive the Cambridge Index of physical activity and questions about daily or weekly fruit and vegetable, whole grain, meat and fish intake, based on the dietary guidelines to lower cardiovascular risk. Objective measures were quantified using accelerometers and plasma carotenoids.

**Results:**

The mean change in response efficacy for physical activity was +0.5 (standard deviation [SD] 2.0) and for diet was +0.5 (SD 2.1).There were no clinically or statistically significant associations between baseline or change in response efficacy and objective and subjective measures of physical activity or objective measures of diet. There was a small statistically significant association between baseline response efficacy and change in self-reported wholegrain consumption, but this is unlikely to be clinically significant.

**Conclusions:**

Response efficacy is not a fundamental determinant of diet and physical activity and should not be the main focus of interventions targeting these behaviours.

## Highlights

• Response efficacy alone is not associated with change in physical activity or diet.
• This was seen for both subjective and objective measures of physical activity and diet.
• Targeting response efficacy alone is unlikely to achieve behaviour change.

## Introduction

Premature morbidity and mortality from non-communicable diseases are significant public health problems.^1^ Numerous observational studies have demonstrated the extent to which modifiable behavioural risk factors, such as physical inactivity and an unhealthy diet, contribute to the aetiology of many non-communicable diseases,^1^, ^2^, ^3^ with dietary risks accounting for 10.8% of all causes of disability-adjusted life years in England and physical activity approximately 3%.^1^

A number of models have been developed to clarify the determinants of health-related behaviours and inform development of interventions. These include the Health Belief Model,^4^, ^5^ Protection Motivation Theory^5^, ^6^ and the Extended Parallel Process Model.^7^, ^8^ One component common to many of these models is response efficacy: the belief that an intervention or action is effective against a perceived health threat^8^ or that changing a behavioural risk factor increases or decreases the risk of developing a disease, for example, increasing physical activity or improving diet reduces the risk of developing cardiovascular disease. Although response efficacy is only one behavioural construct in determining behaviour, understanding the independent role of response efficacy is important when designing public health interventions. There is a large body of evidence reporting an association between response efficacy and intention to change behaviour.^8^, ^9^, ^10^, ^11^ However, the findings are more mixed for associations between response efficacy and behaviour change with some studies reporting a positive association,^8^, ^12^, ^13^ while others do not.^14^, ^15^, ^16^, ^17^ Many of these existing studies include small samples of selected populations, such as young women or undergraduate students,^18^, ^19^, ^20^ and all use self-report measures of behaviour. To our knowledge, there are no studies that report the association between response efficacy and objective measures of behaviour change.

The Information and Risk Modification (INFORM) trial is a randomised controlled trial evaluating the impact of providing phenotypic and genetic coronary heart disease (CHD) risk scores alongside a web-based lifestyle intervention on health-related behaviours, emotional well-being and other CHD-related risk factors.^21^ In addition to subjective measures of behaviour change, it included objective measures of physical activity and diet. In this study, we aimed to quantify the association between response efficacy and change in response efficacy with change in both objectively and subjectively measured physical activity and diet.

## Methods

### Participants

Full details of the design and methods of the INFORM trial have been reported previously.^21^ This present study included data from 953 male and female blood donors for whom information about baseline characteristics was available. Participants in the INFORM trial were recruited as a convenience sample via email invitation of participants in the much larger INTERVAL trial of different intervals between blood donations.^22^ To be included in the INTERVAL study, participants had to be aged ≥18 years, be eligible to donate blood based on the NHS Blood and Transplant guidelines at a permanent NHS Blood and Transplant donor site throughout the trial period and be able to access the internet. Participants in the INFORM trial were aged 40–84 years, able to give informed consent, had no previous medical history of cardiovascular disease, willing to wear a physical activity monitor (accelerometer) for 7 days, willing to provide a blood sample, agreeable to study staff sharing their clinical information with their general practitioner, had a good comprehension of written and verbal English, were not pregnant, had no contraindication to undertaking physical activity and were not enrolled in a clinical trial concerning cardiovascular risk or lifestyle interventions (e.g. diet, physical activity or smoking cessation). To identify those who were enrolled in a clinical trial concerning cardiovascular risk or lifestyle interventions, all participants were asked in the screening and consent form if they were taking part in a clinical trial other than INTERVAL.

The participants were stratified by age (≤60 years) and sex and randomised to the following four groups using a computer programme within the INTERVAL study database:^21^

1. Control group (no lifestyle or risk advice given),
2. Given web-based lifestyle advice only,
3. Given web-based lifestyle advice and phenotypic information on CHD risk,
4. Given web-based lifestyle advice plus phenotypic and genetic information on CHD risk.

### Design

In the present study, data from the four groups were pooled into a prospective cohort and adjustment was made for the trial group and other potential confounders during the analysis. Participants were followed up for 12 weeks.

### Measures

#### Response efficacy

We measured response efficacy at baseline and follow-up (12 weeks after randomisation) using six questions: three relating to physical activity and three relating to diet. Participants were asked how strongly they agreed with the following statements, ‘If I were to be active at a moderate intensity for at least 30 min per day on at least five days a week, I would reduce my risk of having coronary heart disease’, ‘Being physically active is effective in preventing coronary heart disease’, ‘If I am physically active, I am less likely to have coronary heart disease’, ‘If I were to consume five servings of fruit and vegetables each day, I would reduce my risk of having coronary heart disease’, ‘Eating a healthy diet is effective in preventing coronary heart disease’ and ‘If I eat a healthy diet, I am less likely to have coronary heart disease’. Questions were based on Sanderson et al.'s study, which reported a Cronbach's alpha of 0.84,^23^ and were similar to those used in other published studies measuring response efficacy.^24^, ^25^ Responses were on a five-point scale: ‘strongly disagree’; ‘disagree’; ‘not sure’; ‘agree’ and ‘strongly agree’. Responses for physical activity and diet were analysed separately. The scores for each of the three questions were combined to make the range of possible scores for physical activity or diet response efficacy 3–15. Change in response efficacy was calculated by subtracting the response efficacy at baseline from response efficacy at follow-up.

#### Physical activity

Physical activity was measured over 7 days at baseline and follow-up (12 weeks after randomisation) using the wrist-worn Axivity AX3 3-Axis Logging Accelerometer® posted to participants with accompanying instructions. In total, 183 participants had undergone measurement using this device as part of the INTERVAL trial in which case these data were used as baseline measurements for the INFORM trial. Average accelerations (expressed in relative gravity, milligravity [mg]) were measured over the 7-day observation period. Participants were advised that the accelerometer should be worn throughout the day and night, including during washing and bathing, and they should continue their normal activities. We excluded participants who wore the accelerometer for less than 24 h at baseline or follow-up. Change in physical activity was calculated by subtracting the baseline accelerations from the follow-up accelerations.

Self-reported physical activity was assessed at baseline and follow-up using the validated Cambridge Index.^26^ This categorises participants into four groups: ‘inactive’; ‘moderately inactive’; ‘moderately active’ or ‘active’, depending on their responses to two questions based on the levels of occupational physical activity and how many hours spent per week walking, cycling or doing physical exercise such as recreational sports. Individuals with a sedentary occupation were categorised as inactive if they reported zero hours of activity per week, moderately inactive if they reported up to 3.5-h activity per week, moderately active if they reported >3.5 and ≤ 7 h of activity and active if they reported >7 h of activity; individuals with a standing occupation were categorised as moderately inactive if they reported zero hours of activity per week and moderately active or active if they reported ≤3.5 h or >3.5 and ≤ 7 h of activity, respectively; individuals doing manual work were categorised as moderately active if they reported zero hours of activity and active if they reported any activity and those doing heavy manual work were all categorised as active. For this analysis, these categories were treated as if the distance between categories was equal. The change in self-reported physical activity was calculated by subtracting the Cambridge Index at baseline from follow-up.

#### Diet

Diet was measured objectively using plasma carotenoid levels, a proxy measure for fruit and vegetable consumption, one aspect of diet.^27^ The baseline levels of carotenoids were calculated using serum blood samples from the INTERVAL study and at follow-up as part of the INFORM study, 12 weeks after randomisation.^21^

Diet was also assessed by a questionnaire at baseline and follow-up. Diet was divided into fruit and vegetable, wholegrain, meat and fish intake. The questions were developed for the INFORM trial based on the dietary guidelines to lower cardiovascular risk.^28^ Participants were asked how many servings of fruit and vegetables and whole grain they consumed in an average day, and how many servings of meat and fish they ate in a week. Further details of the questions and the available responses are outlined in Appendix 1. Examples of the size of servings per type of food were also included in the questionnaire.

Each type of the subjective diet category was analysed separately, and the results were categorised according to the five responses to each question. For the analysis, these categories were treated as ordinal, with the distance between the categories assumed to be equal. For example, an increase in fruit and vegetable consumption from one per day to two–three per day was considered the same as an increase from two–three to four or from four to five or more. Change in self-reported diet was calculated as the change in the category between baseline and follow-up.

#### Sociodemographic/other covariables

Before randomisation, in the INFORM trial, baseline data relating to age, sex, ethnicity, gross annual income and the highest educational level were collected from participants by a questionnaire.

### Statistical methods

We used linear regression to analyse the associations between baseline response efficacy and change in response efficacy and objective measures of physical activity (accelerations) and diet (carotenoids). At baseline, response efficacy did not conform to the assumptions of linear regression, so we used the squared values. We used ordinal logistic regression to assess the associations between baseline and change in response efficacy and the subjectively measured outcomes. Change in the Cambridge Index and change in consumption of fruit and vegetables, whole grain, meat and fish were categorised into three categories: ‘decrease’; ‘no change’ and ‘increase’. We confirmed agreement with the proportional odds assumption for each model using the ‘omedel’ and ‘brant’ commands in Stata. The coefficients are expressed as odds ratios (ORs) for the ease of interpretation.

The first model for each analysis was a simple regression. The second model adjusted for the a priori confounders: age, sex, income, the highest education level and an original trial group. A *P*-value < 0.05 was considered to be statistically significant. The sample size was fixed by the INFORM study, and retrospective power calculations were not undertaken. If there were more than 10% missing data for an exposure or outcome variable, we compared the baseline characteristics of participants to determine if there was any difference between the two groups. We carried out all statistical analyses using Stata 14.1 statistical software (StataCorp, College Station, Texas, USA).

## Results

The mean age of the participants was 56.7 years (standard deviation [SD] 8.81); 55.7% were men; 91.4% described that their ethnicity as white British; the majority had a university education and a third of the population reported a gross annual income of more than £40,000 (see Table 1).

**Table 1:** Sociodemographic characteristics at baseline (N = 953).

**Characteristic**	**N (%)**^a^
**Age in years [mean (SD)]**	56.7 (8.81)
**Sex**	
Male	531 (55.7)
Female	422 (44.3)
**Ethnicity**	
White, British	784 (91.4)
White, Irish	16 (1.9)
White, other	35 (4.1)
Other ethnicities (Asian, Indian; black, Caribbean; mixed, white and Asian; Asian, other; mixed, white and black Caribbean/African; black, African; Chinese, do not know/prefer not to answer)	23 (2.6)
**Highest education level attained**	
None	8 (0.8)
Primary	3 (0.3)
Secondary	402 (42.3)
University	538 (56.6)
**Gross annual income**	
<£5800	20 (2.1)
£5801–£8000	25 (2.6)
£8001–£40,000	501 (52.9)
>£40,000	321 (33.9)
Do not know	81 (8.5)

SD, standard deviation.

a Unless stated otherwise.

At baseline, the median score for response efficacy was 12 (range 3–15, interquartile range 12–14) for physical activity and 12 (range 3–15, interquartile range 11–13) for diet. The mean change in response efficacy for physical activity was +0.5 (SD 2.0) and for diet, was +0.5 (SD 2.1) [Table 2].

**Table 2:** Change (follow-up minus baseline) in the response efficacy, physical activity and diet.

Variable	Mean change (SD)
**Response efficacy**	
Response efficacy for physical activity	0.5 (2.0)
Response efficacy for diet	0.5 (2.1)
**Physical activity**	
Objective physical activity (mg)	−1.7 (7.6)
Subjective physical activity (Cambridge Index group)	−0.2 (1.0)
**Diet**	
Carotenoids (μmol/L)	−0.5 (1.0)
Self-report fruit and vegetables consumption (category of portions per day)	0.3 (0.8)
Self-report grain consumption (category of portions per day)	0.2 (0.9)
Self-report red meat consumption (category of portions per week)	−0.1 (0.7)
Self-report fish consumption (category of portions per week)	0.1 (0.6)

mg, milligravity; SD, standard deviation.

Overall mean changes in physical activity and diet between baseline and follow-up were small but varied considerably among participants [Table 2]. For physical activity, there was an average mean decrease of 1.7 mg (SD 7.6) for the objective measure (accelerations) and a mean decrease of 0.2 (SD 0.99) for the subjective measure (Cambridge Index group). For diet, there was a mean decrease of 0.5 μmol/L (SD 0.98) in carotenoid levels and for self-reported consumption of fruit and vegetables, grain, meat and fish, there was a mean change of 0.3, 0.2, −0.1 and 0.1 (SD 0.8, 0.9, 0.7, 0.6), respectively.

For change in objectively measured physical activity and diet, 14% and 20% of participants had missing data, respectively. However, there were no significant differences in sociodemographic characteristics among those participants with and without data (data available on request).

There were no associations between baseline squared response efficacy, or change in response efficacy, and change in objectively measured physical activity or diet as shown in Table 3. There were also no associations between response efficacy or change in response efficacy and change in self-reported physical activity as shown in Table 4.

**Table 3:** The association between baseline response efficacy (squared) and change in response efficacy and change in objectively measured physical activity and diet.

Variable	Model 1 (no adjustments)				Model 2 (adjusted for age, sex, income, the highest education level and original trial group)			
	β coefficient (95% CI)	*P*-value	*n*	Adjusted R^2^	β coefficient (95% CI)	*P*-value	*n*	Adjusted R^2^
**Baseline response efficacy**								
Accelerations (mg)	0.0004 (−0.01 to 0.01)	0.95	794	−0.001	−0.0001 (−0.01 to 0.01)	0.98	792	−0.002
Carotenoids (μmol/L)	0.001 (−0.0003 to 0.003)	0.11	762	0.002	0.001 (−0.0001 to 0.003)	0.07	758	0.005
**Change in response efficacy**								
Accelerations (mg)	−0.04 (−0.30 to 0.21)	0.74	753	−0.001	−0.06 (−0.32 to 0.19)	0.62	751	0.002
Carotenoids (μmol/L)	−0.02 (−0.05 to 0.02)	0.34	726	−0.001	−0.02 (−0.05 to 0.02)	0.34	723	0.008

CI, confidence interval; mg, milligravity.

**Table 4:** The association between baseline response efficacy and change in response efficacy and change in self-reported physical activity (Cambridge Index) and diet.

Variable	Model 1 (no adjustments)			Model 2 (adjusted for age, sex, income, highest education level and original trial group)		
	Odds ratio (95% CI)	*P*-value	*n*	Odds ratio (95% CI)	*P*-value	*n*
**Baseline response efficacy**						
Change in Cambridge Index	1.01 (0.94–1.08)	0.71	945	0.99 (0.92–1.07)	0.88	940
Change in fruit and vegetable consumption	1.06 (0.98–1.13)	0.13	856	1.03 (0.97–1.12)	0.31	852
Change in wholegrain consumption	1.10 (1.03–1.18)	0.004†	856	1.10 (1.02–1.17)	0.008†	852
Change in meat consumption	0.99 (0.918–1.07)	0.76	856	1.01 (0.93–1.08)	0.90	852
Change in fish consumption	1.01 (0.94–1.10)	0.71	856	1.00 (0.93–1.09)	0.82	852
**Change in response efficacy**						
Change in Cambridge Index	1.02 (0.95–1.10)	0.62	856	1.03 (0.95–1.10)	0.52	852
Change in fruit and vegetable consumption	1.06 (0.99–1.13)	0.07	856	1.05 (0.99–1.12)	0.11	852
Change in wholegrain consumption	0.99 (0.93–1.05)	0.64	856	0.98 (0.92–1.04)	0.48	852
Change in meat consumption	0.97 (0.91–1.03)	0.34	856	0.97 (0.91–1.04)	0.38	852
Change in fish consumption	1.04 (0.97–1.11)	0.30	856	1.02 (0.96–1.10)	0.49	852

CI, confidence interval.

†*P* < 0.05.

However, for self-reported diet, there was a statistically significant association between baseline response efficacy and change in wholegrain consumption (OR: 1.10, 95% confidence interval: 1.03 to 1.18, *P*-value 0.005) but no associations between the change in response efficacy and change in self-reported diet, as shown in Table 4.

## Discussion

### Main findings

To our knowledge, this is the first study that examines the association between response efficacy and objectively measured physical activity or diet. In this large cohort, there were no clinically or statistically significant associations between response efficacy and objectively or subjectively measured physical activity. There was a small association between baseline response efficacy and change in self-reported wholegrain consumption. However, the other associations between response efficacy and diet, both reported and objectively measured, were small and not clinically significant.

### Comparison with existing literature

It is difficult to directly compare the baseline response efficacy or change in response efficacy found in this study with the published literature as there is no universal method of measurement. Of the studies that had used a similar scale,^14^, ^15^, ^25^, ^29^ most had mean baseline response efficacy scores higher than in this study (ranging from 4.05 to 4.66 for individual questions), and none reported changes in response efficacy.

The finding in this study of no association between response efficacy or change in response efficacy and subjectively or objectively measured physical activity is contrary to the systematic review by Bui et al. in which response efficacy was positively correlated with self-reported intention and behaviour.^12^ However, in that review, the authors only included studies that investigated the association between the components of the Protection Motivation Theory and subjectively measured specific aspects of physical activity, for example strength and balance training, workplace-based activity and vigorous physical activity sessions. This discrepancy may, therefore, be explained by the more general questions used in that study around habitual rather than specific behaviour and the additional use of objective measures that are more robust than the measures of intention. Thirteen of the 18 studies that measured response efficacy and were included in the review by Bui et al. also included selected groups, such as university psychology students or predominantly female study groups.^16^, ^18^, ^30^ A further explanation for the limited number of studies reporting no association between response efficacy and behaviour may be publication bias.

We are aware of only one previous study that investigated the association between response efficacy and dietary outcomes.^29^ That study used self-reported measures of a low-fat diet and path analysis of the Protection Motivation Theory and found positive associations between response efficacy and reported low-fat diet. We did not measure the consumption of low-fat food in our study but instead found a small significant association between response efficacy and reported wholegrain consumption and no associations for other aspects of self-reported diet or carotenoid levels.

### Strengths and limitations

The main strengths of this study are the large population size and objective measures used to assess the change in behaviour which minimises the risk of bias and improves precision. The participants in this cohort also had a wider age range and more equal sex distribution compared with previous studies that recruited university students or mainly female participants.^15^, ^16^, ^18^, ^30^ This makes the findings more generalisable to the population. However, the study population was drawn from a convenience sample of blood donors, who are likely to be healthier (owing to the extensive exclusion criteria for donating blood) and potentially more interested in their own health than the general population. Although the levels of physical activity were similar to other population-based studies using the same type of accelerometer,^31^ the mean levels of serum carotenoids for participants in the INFORM study were slightly higher than those in other studies.^32^, ^33^ It is, therefore, possible that there may have been a ‘ceiling’ to how much healthier their lifestyle could become, constraining the likelihood of further changes in diet and physical activity during this study. The lack of association between response efficacy and behaviour change may, therefore, be due to a lack of change in physical activity and diet across the whole study cohort.

The use of objective measures of outcomes reduced recall bias and response bias, and the use of accelerometers continuously in a free-living situation increased the number of measurements and reduced the risk of random error. However, not all types of movement can be captured. Carotenoids have a strong correlation with fruit and vegetable intake, so are a good objective measure of these aspects of diet^27^ but not, for example, the consumption of whole grains. Our finding of an association with wholegrain consumption could also be the result of multiple hypotheses testing so should be interpreted with caution.

There is also no ‘gold standard’ measurement for the latent variable response, making any measurement scale difficult to validate. However, the questions used in this study have been used previously,^23^, ^29^ and the impact of response bias was minimised by using the same questionnaire at baseline and follow-up.

By focussing on response efficacy alone, we have also only investigated one aspect in a complex system of determinants of behaviour change and cannot exclude an interaction between response efficacy and other behaviour change constructs. Given the lack of evidence in this area on response efficacy alone, however, this study provides an important contribution to the literature on the independent role of response efficacy in changing behaviour.

### Implications for practice, policy and research

There is a growing burden of preventable chronic conditions associated with unhealthy diets and inactivity. The development of effective strategies to influence these behavioural determinants at the individual and collective level is, therefore, a priority for policymakers. We have shown that response efficacy and change in response efficacy alone are not associated with change in behaviour. Consequently, interventions that seek to influence the behaviour solely by targeting response efficacy are unlikely to be effective. Perhaps, instead interventions might target other behaviour change components. For example, there is a substantial body of evidence of associations between self-efficacy and change in behaviour using both subjective and objective measures of behaviour change.^34^, ^35^, ^36^ Alternatively, interventions based on frameworks incorporating multiple elements targeting multiple behaviour change techniques, such as the Behaviour Change Wheel developed by Michie et al.,^37^ may be more effective than those targeting individual variables from specific psychological models. There is also good evidence that habitual behaviours such as diet and physical activity are subject to powerful environmental influence rather than the result of a conscious cognitive decision-making process.^38^, ^39^ Consequently, there is growing interest in the potential for alterations to the environment, from the size of wine glasses to the introduction of cycle lanes, to positively influence health behaviours. Other collective approaches that have proved effective include taxes on sugar-sweetened beverages in Mexico.^40^ Such population-based approaches to shifting the distribution of behaviours may be effective when used alone, as demonstrated in North Karelia,^41^ or in conjunction with individual-based approaches, such as in the Västerbotten Intervention Programme that incorporated individual cardiovascular risk assessment and population-wide strategies.^42^

Whether a population or individual-based approach for behaviour change is evaluated, research using sample populations that are due to be targeted by interventions is much needed. In addition, this research should incorporate precise objective measures, which is lacking in the current evidence.

In summary, we have identified no association between response efficacy and change in health behaviours and suggest that targeting response efficacy alone is unlikely to achieve behaviour change.

## Author statements

### Acknowledgements

A complete list of investigators and contributors to the INTERVAL trial is provided by Silarova et al.^21^ A complete list of investigators and contributors to the INFORM study is provided by Moore et al.^22^ NHS Blood and Transplant, the sponsor of the INTERVAL trial, assisted with the involvement of blood donors in INFORM. The web-based lifestyle intervention for CHD prevention is based on one that was originally developed for the Heart to Health study (http://hpdp.unc.edu/research/projects/heart-to-health/). In addition, some materials were originally developed by Leicester Diabetes Centre (http://www.leicesterdiabetescentre.org.uk/). Affymetrix (Santa Clara, US) provided genotyping. The MRC Epidemiology Unit Physical Activity Team provided expertise. UK Biocentre (Stockport, UK) provided laboratory support. Vitas (Oslo, Norway) conducted assays. The authors would like to thank patient and public involvement representatives—Kathryn Lawrence and Chris Girling for reviewing study documents. Finally, they would like to thank to all participants who agreed to take part in qualitative part of INFORM trial.

### Ethical approval

All procedures performed in studies involving human participants were in accordance with the ethical standards of the institutional and/or national research committee and with the 1964 Helsinki Declaration and its later amendments or comparable ethical standards. NRES Committee East of England–Cambridge Central (14/EE/1164) granted ethical approval for the INFORM study on 3/12/2014. NHS Research and Development assurance was given by Cambridgeshire and Peterborough CCG and CRN: Eastern. The trial was registered at the ISRCTN Registry (ISRCTN17721237) on 12/01/2015.

### Funding

The INFORM study was funded by European Commission Framework 7 EPIC-CVD Grant agreement (No. 279233). NHS Blood and Transplant funded the INTERVAL trial. Deoxyribonucleic acid extraction and genotyping in INTERVAL/INFORM was funded by the United Kingdom National Institute of Health Research. The coordinating team for INTERVAL/INFORM at the Cardiovascular Epidemiology Unit of the University of Cambridge was supported by core funding from the United Kingdom Medical Research Council (G0800270), British Heart Foundation (SP/09/002), British Heart Foundation Cambridge Cardiovascular Centre of Excellence and United Kingdom National Institute for Health Research Cambridge Biomedical Research Centre. J.A.U.-S. was funded by a National Institute for Health Clinical Lectureship, and B.S. was supported by the Medical Research Council (MC_UU_12015/4). The University of Cambridge has received salary support in respect of SJG from the NHS in the East of England through the Clinical Academic Reserve.

### Competing interests

The authors declare that they have no conflict of interests.

### Informed consent

Informed consent was obtained from all individual participants included in the study.

### Disclaimer of views

The views expressed in this publication are those of the authors and not necessarily those of the NHS, the National Institute for Health Research, or the Department of Health.

## Appendix 1 Questions assessing self-reported diet

For the following questions please tick the box that applies to you the most.

How many servings of fruit and vegetables do you consume on an average day?

(one serving is equivalent to 1 piece of fruit or a standard glass [200 mls] of unsweetened juice [only one glass counts]; potatoes are not included as a vegetable).

• None
• One a day
• 2–3 per day
• 4 per day
• 5 or more per day

How many servings of whole grains do you consume on an average day? (one serving of whole grain foods include: whole meal or whole grain bread [1 slice]; wholewheat pasta [2 tablespoons cooked]; wholegrain breakfast cereal [3 tablespoons] or whole rolled porridge oats [1 tablespoon uncooked]).

• None
• One a day
• 2 per day
• 3–4 per day
• 5 or more per day

How many servings of red meat (beef, lamb, pork, bacon, ham, sausages and burgers) do you consume during an average week? (one serving is equivalent to 80 g, which is approximately the size of a pack of cards; 2 rashers of bacon or 1 ½ standard size sausages).

Some average serving sizes of red meat (raw weight): medium steak = 145 g (5.1 oz); pork chop = 75 g (2.6 oz); spaghetti Bolognese with minced beef = 140 g (4.9 oz); serving of roast beef = 90 g (3.2 oz).

• None
• One a week
• 2–3 per week
• 4–6 per week
• 7 or more per week

How many servings of fish do you consume during an average week?

(one serving is equal to 140 g of cooked fish, or equivalent in size to approximately the size and thickness of the palm of your hand).

• None
• One a week
• 2–4 per week
• 5–6 per week
• 7 or more per week

## References

[1] Newton J.N., Briggs A.D.M., Murray C.J.L., Dicker D., Foreman K.J., Wang H. (2015). Changes in health in England, with analysis by English regions and areas of deprivation, 1990–2013: a systematic analysis for the Global Burden of Disease Study 2013. Lancet.

[2] D'Agostino R.B., Vasan R.S., Pencina M.J., Wolf P.A., Cobain M., Massaro J.M. (2008 Feb 12). General cardiovascular risk profile for use in primary care: the Framingham Heart Study. Circulation [Internet].

[3] Muller D.C., Murphy N., Johansson M., Ferrari P., Tsilidis K.K., Boutron-Ruault M.-C. (2016). Modifiable causes of premature death in middle-age in Western Europe: results from the EPIC cohort study. BMC Med.

[4] Janz N.K., Becker M.H. (1984). The health belief model: a decade later. Health Educ Q.

[5] Sutton S. (2015). Health behavior, psychosocial Theories of. Int Encycl Soc Behav Sci [Internet].

[6] Rogers R.W., Cacioppo J., Petty R. (1983). Cognitive and physiological processes in fear appeals and attitude change: a revised theory of protection motivation. Social psychophysiology: a source book.

[7] Peters G.J.Y., Ruiter R.A.C., Kok G. (2012). Threatening communication: a critical re-analysis and a revised meta-analytic test of fear appeal theory. Health Psychol Rev.

[8] Witte K., Allen M. (2000). A meta-analysis of fear appeals: implications for effective public health campaigns. Health Educ Behav.

[9] Floyd D.L., Prentice-Dunn S., Rogers R.W. (2000). A meta-analysis of research on protection motivation theory. J Appl Soc Psychol.

[10] Hoog N.De, De Wit J.B.F. (2003). The impact of fear appeals on processing. Pers Soc Psychol Bull.

[11] Ruiter R.A.C., Kessels L.T.E., Peters G.-J.Y., Kok G. (2014). Sixty years of fear appeal research: current state of the evidence. Int J Psychol.

[12] Bui L., Mullan B., McCaffery K. (2013 Jan). Protection motivation theory and physical activity in the general population: a systematic literature review. Psychol Health Med [Internet].

[13] Fu L.Y., Bonhomme L.-A., Cooper S.C., Joseph J.G., Zimet G.D. (2014). Educational interventions to increase HPV vaccination acceptance: a systematic review. Vaccine.

[14] Lippke S., Plotnikoff R.C. (2009). The protection motivation theory within the stages of the transtheoretical model – stage-specific interplay of variables and prediction of exercise stage transitions. Br J Health Psychol.

[15] Rhodes R.E., Plotnikoff R.C., Courneya K.S. (2008). Predicting the physical activity intention-behavior profiles of adopters and maintainers using three social cognition models. Ann Behav Med.

[16] Tavares L.S., Plotnikoff R.C., Loucaides C. (2009). Social-cognitive theories for predicting physical activity behaviours of employed women with and without young children. Psychol Heal Med.

[17] Wurtele S.K. (1988). Increasing women's calcium intake: the role of health beliefs, intentions, and health value. J Appl Soc Psychol.

[18] Courneya K.S., Hellsten L.A.M. (2001). Cancer prevention as a source of exercise motivation: an experimental test using protection motivation theory. Psychol Health Med.

[19] Hodgkins S., Orbell S. (1998). Can protection motivation theory predict behaviour? A longitudinal test exploring the role of previous behaviour. Psychol Health.

[20] Stanley M., Maddux J. (1986). Cognitive processes in health enhancement: investigation of a combined protection motivation and self-efficacy model. Basic Appl Soc Psych.

[21] Silarova B., Lucas J., Butterworth A.S., Di Angelantonio E., Girling C., Lawrence K. (2015). Information and Risk Modification Trial (INFORM): design of a randomised controlled trial of communicating different types of information about coronary heart disease risk, alongside lifestyle advice, to achieve change in health-related behaviour. BMC Publ Health.

[22] Moore C., Sambrook J., Walker M., Tolkien Z., Kaptoge S., Allen D. (2014 Sep 17). The INTERVAL trial to determine whether intervals between blood donations can be safely and acceptably decreased to optimise blood supply: study protocol for a randomised controlled trial. Trials [Internet].

[23] Sanderson S.C., Persky S., Michie S. (2010). Psychological and behavioral responses to genetic test results indicating increased risk of obesity: does the causal pathway from gene to obesity matter?. Publ Health Genom.

[24] Plotnikoff R.C., Higginbotham N. (2002). Protection motivation theory and exercise behaviour change for the prevention of coronary heart disease in a high-risk, Australian representative community sample of adults. Psychol Health Med.

[25] Plotnikoff R.C., Rhodes R.E., Trinh L. (2009). Protection motivation theory and physical activity: a longitudinal test among a representative population sample of Canadian adults. J Health Psychol.

[26] Wareham N.J., Jakes R.W., Rennie K.L., Schuit J., Mitchell J., Hennings S. (2003). Validity and repeatability of a simple index derived from the short physical activity questionnaire used in the European Prospective Investigation into Cancer and Nutrition (EPIC) study. Publ Health Nutr.

[27] Baldrick F.R., Woodside J.V., Elborn J.S., Young I.S., Mckinley M.C. (2011). Critical reviews in food science and nutrition biomarkers of fruit and vegetable intake in human intervention studies: a systematic review biomarkers of fruit and vegetable intake in human intervention studies: a systematic review. Crit Rev Food Sci Nutr.

[28] JBS 3 Board (2014). Joint British Societies' consensus recommendations for the prevention of cardiovascular disease. Heart.

[29] Plotnikoff R.C., Higginbotham N. (1995). Predicting low-fat diet intentions and behaviors for the prevention of coronary heart disease: an application of protection motivation theory among an Australian population. Psychol Health.

[30] Graham S.P., Prapavessis H., Cameron L.D. (2006). Colon cancer information as a source of exercise motivation. Psychol Health.

[31] Doherty A., Jackson D., Hammerla N., Plotz T., Olivier P., Granat M.H. (2017). Large scale population assessment of physical activity using wrist worn accelerometers: the UK biobank study. PLoS One.

[32] Burrows T.L., Williams R., Rollo M., Wood L., Garg M.L., Jensen M. (2015). Plasma carotenoid levels as biomarkers of dietary carotenoid consumption: a systematic review of the validation studies. J Nutr Intermed Metab.

[33] Hofe C., Feng L., Zephyr D., Stromberg A., Hennig B., Gaetke L. (2014). Fruit and vegetable intake, as reflected by serum carotenoid concentrations, predicts reduced probability of PCB-associated risk for type 2 diabetes: NHANES 2003–2004. Nutr Res.

[34] Palmeira A., Teixeira P., Branco T., Martins S., Minderico C., Barata J. (2007). Predicting short-term weight loss using four leading health behavior change theories. Int J Behav Nutr Phys Act.

[35] Strecher V.J., McEvoy DeVellis B., Becker M.H., Rosenstock I.M. (1986). The role of self-efficacy in achieving behavior change health. Health Educ Q.

[36] Wingo B., Desmond R., Brantley P., Appel L., Svetkey L., Stevens V. (2013). Self-efficacy as a predictor of weight change and behavior change in the PREMIER trial brooks. J Nutr Educ Behav.

[37] Michie S., van Stralen M.M., West R. (2011). The behaviour change wheel: a new method for characterising and designing behaviour change interventions. Implement Sci.

[38] Morland K., Diez Roux A.V., Wing S. (2006). Supermarkets, other food stores, and obesity: the atherosclerosis risk in communities study. Am J Prev Med.

[39] Papas M.A., Alberg A.J., Ewing R., Helzlsouer K.J., Gary T.L., Klassen A.C. (2007). The built environment and obesity. Epidemiol Rev.

[40] Colchero M.A., Popkin B.M., Rivera J.A., Ng S.W. (2016). Beverage purchases from stores in Mexico under the excise tax on sugar sweetened beverages: observational study. BMJ.

[41] Puska P. (2002). Successful prevention of non-communicable diseases: 25 year experiences with North Karelia Project in Finland. Publ Heal Med.

[42] Norberg M., Wall S., Boman K., Weinehall L. (2010). The Västerbotten Intervention Programme: background, design and implications. Glob Health Action.

